# Terminal Parent Phosphanide and Phosphinidene Complexes of Zirconium(IV)

**DOI:** 10.1002/anie.201703870

**Published:** 2017-05-26

**Authors:** Hannah Stafford, Thomas M. Rookes, Elizabeth P. Wildman, Gábor Balázs, Ashley J. Wooles, Manfred Scheer, Stephen T. Liddle

**Affiliations:** ^1^ School of Chemistry The University of Manchester Oxford Road Manchester M13 9PL UK; ^2^ Institute of Inorganic Chemistry University of Regensburg Universitätsstrasse 31 93053 Regensburg Germany

**Keywords:** agostic-type interactions, phosphanides, phosphinidenes, X-ray crystallography, zirconium

## Abstract

The reaction of [Zr(Tren^DMBS^)(Cl)] [**Zr1**; Tren^DMBS^=N(CH_2_CH_2_NSiMe_2_Bu^*t*^)_3_] with NaPH_2_ gave the terminal parent phosphanide complex [Zr(Tren^DMBS^)(PH_2_)] [**Zr2**; Zr−P=2.690(2) Å]. Treatment of **Zr2** with one equivalent of KCH_2_C_6_H_5_ and two equivalents of benzo‐15‐crown‐5 ether (B15C5) afforded an unprecedented example (outside of matrix isolation) of a structurally authenticated transition‐metal terminal parent phosphinidene complex [Zr(Tren^DMBS^)(PH)][K(B15C5)_2_] [**Zr3**; Zr=P=2.472(2) Å]. DFT calculations reveal a polarized‐covalent Zr=P double bond, with a Mayer bond order of 1.48, and together with IR spectroscopic data also suggest an agostic‐type Zr⋅⋅⋅HP interaction [∡_ZrPH_=66.7°] which is unexpectedly similar to that found in cryogenic, spectroscopically observed phosphinidene species. Surprisingly, computational data suggest that the Zr=P linkage is similarly polarized, and thus as covalent, as essentially isostructural U=P and Th=P analogues.

Well‐defined transition‐metal phosphinidene complexes (L_n_M=PR) are of interest owing to a desire to better understand their bonding and PR‐group transfer chemistry.^1^, ^2^ However, although such complexes were first reported three decades ago,^3^ they remain a relatively rare class of metal‐ligand multiple bond. This relative paucity reflects the inherent nature of the phosphinidene functional group, which as a free moiety is very reactive due to the P‐triplet ground state and unsaturated valence shell.^4^ Stabilization of a phosphinidene by metal‐coordination is an attractive strategy,^1^ but normally also demands a sterically bulky group at phosphorus to kinetically stabilize the M=PR linkage. Indeed, it is notable that under ambient conditions all isolable transition‐metal phosphinidene complexes exhibit sterically demanding R groups to kinetically protect these vulnerable M=PR bonds;^3^, ^5^, ^6^, ^7^, ^8^, ^9^ in a broader sense the only exceptions are where fundamental, elegant species such as H_2_M=PH (M=Ti, Zr, and Hf) have been prepared and spectroscopically observed under cryogenic conditions.^10^ Early transition‐metal phosphinidene complexes are perhaps the most developed of all metal‐phosphinidenes, so it is surprising that an early transition‐metal parent phosphinidene has not yet been realized under ambient conditions.

Recently, as part of our work on actinide‐ligand multiple bonds,^11^ we reported uranium and thorium phosphinidene complexes using the parent phosphinidene (HP)^2−^,^12^ despite the large triplet–singlet energy gap of approximately 22 kcal mol^−1^ for free PH,4g which had previously only been seldom observed as a fleeting spectroscopic intermediate or probed theoretically.^4^ Those two actinide complexes are the only two M=PH complexes yet isolated outside cryogenic spectroscopic experiments, and were supported by the very sterically demanding triamidoamine ligand N(CH_2_CH_2_NSiPr^*i*^_3_)_3_ (Tren^TIPS^). Noting that it is unusual for key metal–ligand linkages to be realized in d‐block chemistry subsequent to f‐block ones, rather than the other way around, we wondered whether Group 4 analogues could be prepared, which would provide a basis from which to make d–f bonding comparisons with a phosphinidene moiety that remains exceedingly rare under any circumstance.

Herein, we report a terminal parent zirconium‐phosphinidene, which represents the first example of a structurally authenticated transition‐metal complex of the parent phosphinidene group. Despite very different zirconium coordination environments, an agostic‐type Zr⋅⋅⋅HP interaction is found in the phosphinidene complex reported herein as has been suggested from matrix isolation data on H_2_Zr=PH.^10^ Surprisingly, quantum chemical calculations suggest that the Zr=P bond reported herein is nearly as covalent as essentially isostructural U=P and Th=P bonds. This is contrary to expectations, given the general view that d‐block metals, on a like‐for‐like basis, should be expected to engage in more covalent bonding than f‐block elements.

According to Pyykkö, the covalent single bond radius of Zr (1.54 Å) is 0.16 Å smaller than that of U (1.70 Å).^13^ We thus surmised that a less sterically demanding ligand than Tren^TIPS^ would be required, so we chose N(CH_2_CH_2_NSiMe_2_Bu^*t*^)_3_ (Tren^DMBS^). Accordingly, treatment of [Zr(Tren^DMBS^)(Cl)] (**Zr1**)^14^ with NaPH_2_ 15 afforded, after work‐up and recrystallization, yellow crystals of [Zr(Tren^DMBS^)(PH_2_)] (**Zr2**) in 55 % isolated yield, Scheme 1. The synthesis of **Zr2** is notable in that, unlike U and Th congeners,^12^ it does not require a [M(Tren)][BPh_4_] separated ion‐pair formulation to install the pnictide. The ^1^H NMR spectrum of **Zr2** is consistent with a pseudo‐*C*_3_ symmetric species, and the phosphanide hydrogen atoms resonate as a doublet centered at 1.63 ppm (*J*_PH_=166.6 Hz); the ^31^P NMR spectrum likewise exhibits a triplet centered at −175.3 ppm. The ATR‐IR spectrum of **Zr2** exhibits a broad feature at approximately 2288 cm^−1^, which is a composite of two overlapping absorptions; this compares well to computed stretching frequencies of 2313 and 2332 cm^−1^ from an analytical frequencies calculation.

**Scheme 1:**
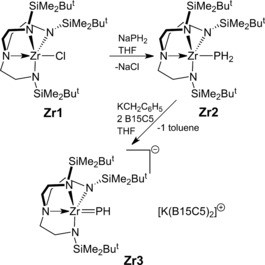
Synthesis of **Zr2** and **Zr3** from **Zr1**. B15C5=benzo‐15‐crown‐5 ether.

The solid‐state structure of **Zr2** was confirmed by X‐ray crystallography (see Supporting Information) which revealed a Zr–P distance of 2.690(2) Å; this is slightly longer than the sum of the single bond covalent radii of Zr and P (2.65 Å),^13^ but shorter than the Zr−P distance of 2.725(2) Å in [Zr{N(CH_2_CH_2_NSiMe_3_)_3_}(PHPh)]^16^ where the phosphanide may be a weaker donor from charge delocalization into the P‐phenyl ring.

Treatment of **Zr2** with one and two molar equivalents of KCH_2_Ph and benzo‐15‐crown‐5 ether (B15C5), respectively, afforded the zirconium terminal parent phosphinidene complex [Zr(Tren^DMBS^)(PH)][K(B15C5)_2_] (**Zr3**), isolated as orange crystals in 24 % yield after work‐up and recrystallization. ^1^H NMR spectroscopy of freshly prepared reaction mixtures suggests that **Zr3** is the major product, and so the low crystalline yield reflects the high solubility and inherently oily nature of this separated ion pair. However, we note that **Zr3** is clearly on the cusp of stability, because its solutions in aromatic solvents completely decompose to unknown products within hours. Like **Zr2**, the ^1^H NMR spectrum of **3** is indicative of a pseudo‐*C*_3_ symmetric zirconium species, and the phosphinidene hydrogen resonates as a doublet at 8.53 ppm (*J*_PH_=173.4 Hz). The ^31^P NMR spectrum of **Zr3** exhibits a broad resonance at +246.75 ppm, shifted approximately 422 ppm from that of **Zr2**, where the P−H coupling is obscured by the broad linewidth (full‐width at half maximum=256 Hz). The ATR‐IR spectrum of **Zr3** exhibits one very broad and thus overall weak absorption, tentatively attributed to the P−H stretch at approximately 2100 cm^−1^, which compares well to a calculated P−H stretching frequency of 2140 cm^−1^ from an analytical frequencies calculation.

The solid‐state molecular structure of **Zr3** was determined by X‐ray crystallography, Figure 1, confirming the separated ion pair nature, and thus terminal phosphinidene assignment of **Zr3**. The Zr=P distance is found to be 2.4723(17) Å, which represents a contraction of around 0.22 Å (ca. 9 %) compared to **Zr2**. However, the Zr=P distance in **Zr3** is longer than the sum of the covalent double‐bond radii of Zr and P of 2.29 Å;^13^ this may be due to the electron‐rich anionic formulation of the phosphinidene portion of **Zr3**, and/or due to the phosphinidene being located *trans* to the trialkyl‐amine donor of the Tren^DMBS^ ligand. Two observations consistent with those notions are that the Zr−N_amine_ distance in **Zr2** (2.516(5) Å) is shorter than the corresponding distance in **Zr3** (2.586(4) Å), and the Zr−N_amide_ distances in **Zr3** (average of 2.124(8) Å) are considerably longer than the analogous distances in **Zr2** (2.062(9) Å). However, the Zr=P distance in **Zr3** is shorter than the Zr=P distance in [Zr(η^5^‐C_5_H_5_)_2_(P{2,4,6‐Bu^*t*^_3_C_6_H_2_})(PMe_3_)] (2.505(4) Å),5q and compares well to the computed Zr=P distance of 2.324 Å in H_2_Zr=PH,^10^ which is considerably less sterically encumbered, has a lower coordination number at Zr than in **Zr3**, and is also neutrally charged overall. Interestingly, although there are no obvious interactions between the phosphanide hydrogen atoms and zirconium center in **Zr2**, in **Zr3** the phosphinidene hydrogen, located and refined by a combination of crystallographic difference Fourier map data and DFT calculations, appears to be engaged in a weak agostic‐type Zr⋅⋅⋅HP interaction (Zr⋅⋅⋅H=2.322(19) Å; ∡_ZrPH_=66.7(8)°). The ∡_ZrPH_ for **Zr3** is very similar to that computed for H_2_Zr=PH (∡_ZrPH_=63.8°), despite the very different zirconium coordination numbers and geometries of the two complexes.^10^ The Zr⋅⋅⋅H distance in **Zr3** is considerably longer than the sum of the single bond covalent radii of Zr and H (1.86 Å),^13^ and the analogous Zr⋅⋅⋅H distance of 2.13 Å in H_2_Zr=PH,^10^ which can be rationalized by the aforementioned steric and charge differences between **Zr3** and H_2_Zr=PH.^10^

**Figure 1:**
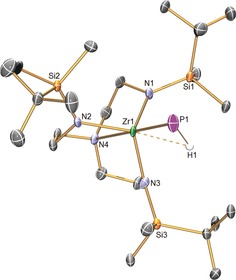
Molecular structure of **Zr3** at 150 K. Thermal ellipsoids set at 30 % probability, and non‐phosphorus‐bound hydrogen atoms, minor disorder components, and the potassium bis(B15C5) cation component, are omitted for clarity. The Zr⋅⋅⋅HP interaction is represented by a dashed line. Selected bond lengths [Å] and angles [°]: Zr1−P1 2.4723(17), Zr1−N1 2.135(5), Zr1−N2 2.109(5), Zr1−N3 2.127(5), Zr1−N4 2.586(4), Zr⋅⋅⋅HP 2.322(19); Zr1−P1−H1 66.7(8).

In order to gain a greater understanding of the nature of the bonding in the Zr=PH unit in **Zr3**, we carried out DFT calculations on the full anion component of **Zr3**, **Zr3^−^**, and for comparison the full molecule of **Zr2**, Table 1. The geometry‐optimized gas‐phase structures of **Zr2** and **Zr3^−^** closely match the experimental solid‐state structures, to within 0.05 Å and 2° of corresponding bond lengths and angles, and so we conclude that the calculations provide a qualitative picture of the electronic structures of these molecules. In order to make wider comparisons, we also compile the computed data for the closely related phosphinidene anions [U(Tren^TIPS^)(PH)]^−^ (**U3^−^**) and [Th(Tren^TIPS^)(PH)]^−^ (**Th3^−^**) in Table 1.^12^

**Table 1:** Selected computed DFT, NBO, and QTAIM data for **Zr2**, **Zr3^−^**, **U3^−^**, and **Th3^−^**.

	Bond lengths and indices		MDC atomic charges		NBO σ‐component^[f]^			NBO π‐component^[f]^			QTAIM parameters^[g]^			
Entry^[a]^	M‐P^[b]^	BI^[c]^	*q_M_* ^[d]^	*q*_P_^[e]^	%M	%P	M s:p:d:f	%M	%P	M s:p:d:f	*ρ*(*r*)	∇^2^ *ρ*(*r*)	*H*(*r*)	*ϵ*(*r*)
**Zr2**	2.739	0.83	1.50	−0.22	10	90	15:0:85:0	–	–	–	0.05	0.05	−0.01	0.06
**Zr3^‐^**	2.473	1.48	1.24	−0.51	22	78	18:0:82:0	29	71	0:0:100:0	0.08	0.08	−0.03	0.27
**U3^‐^**	2.621	1.92	2.32	−1.16	24	76	0:0:20:80	28	72	0:1:30:69	0.08	0.07	−0.03	0.20
**Th3^‐^**	2.709	1.67	2.24	−0.91	12	88	4:0:44:52	14	86	0:1:54:45	0.07	0.06	−0.02	0.40

[a] All molecular geometries optimized without symmetry constraints at the LDA VWN BP TZP/ZORA level. [b] Calculated M−P distances (Å). [c] Mayer bond indices. [d] MDC‐q charges on metal atoms. [e] MDC‐q charges on phosphorus atoms. [f] Natural Bond Orbital (NBO) analyses. [g] QTAIM (atoms in molecules) topological electron density [*ρ*(*r*)], Laplacian [∇^2^ *ρ*(*r*)], electronic energy density [*H*(*r*)], and ellipticity [*ϵ(r)*] bond‐critical‐point data.

As expected, the Zr−P bond order virtually doubles upon moving from phosphanide **Zr2** to phosphinidene **Zr3^−^**. For comparison, the Zr−N_amide_/Zr−N_amine_ bond orders are 0.68/0.22 and 0.53/0.14 for **Zr2** and **Zr3^−^**, respectively, which suggests that as the Zr=P double bond develops, the Zr−N interactions diminish, as suggested by the crystallographic data. Likewise, the charge on Zr decreases as the Zr=P bond is established, and the negative charge on P increases more than two times, in line with the formal mono‐ and di‐anionic charges on H_2_P^−^ and HP^2−^, respectively.

Inspection of the Kohn–Sham molecular orbitals (KSMOs) of **Zr3^−^** reveals the anticipated Zr=P π (HOMO) and σ (HOMO−1) bonds, Figure 2. Unfortunately, these KSMOs are mixed with amide N‐lone pair orbital coefficients. Therefore, in order to inspect the Zr=P double bond in isolation we used NBO (natural bond orbital) analysis, which also finds discrete Zr=P σ‐ and π‐bonding combinations. On moving from **Zr2** to **Zr3^−^** the Zr contributions to the Zr−P bonds increase markedly, and although there is no π‐bonding combination to compare between **Zr2** and **Zr3^−^** the Zr contribution to the Zr−P σ bond more than doubles on moving from the former to the latter.

**Figure 2:**
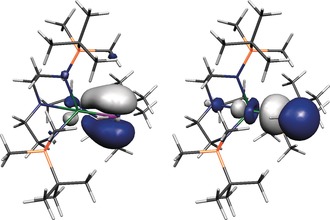
Kohn–Sham frontier molecular orbitals representing the principal components of the Zr=P double‐bond interaction in **Zr3^−^**. Left: HOMO (164, −0.511 eV). Right: HOMO−1 (163, −0.849 eV).

To gain further insight into the nature of the Zr=P bond in **Zr3^−^**, we used QTAIM (atoms‐in‐molecules) to analyze the Zr=P bond topology. This reveals, despite a superficial picture of covalence from the orbital based methods, a more ionic bonding picture, where the *ρ*(*r*) term is in the region typically associated with predominantly ionic bonding (<0.1). However, the presence of spherical and non‐spherical electron density, with respect to density along the Zr−P internuclear vector, for **Zr2** and **Zr3^−^**, respectively, is consistent with the presence of formal single‐ and double‐bonding interactions in these complexes, though of course in **Zr3^−^** the Zr^+^−P^−^ dipolar resonance form will contribute to the bonding overall.^17^

The significantly smaller P−H stretching frequency of **Zr3** compared to **Zr2**, along with crystal structure data for **Zr3**, are certainly suggestive of an agostic‐type Zr⋅⋅⋅HP interaction in **Zr3**.^10^ Interestingly, the DFT calculations return a Zr−H bond order of 0.14 in support of an agostic‐type Zr⋅⋅⋅HP interaction. However, close inspection of the QTAIM data does not reveal a Zr−H bond‐critical point in **Zr3^−^**, and so although the presence of a weak agostic‐type Zr⋅⋅⋅HP interaction is likely, it is not unequivocally confirmed.

The ionic‐bonding picture of **Zr3^−^** is perhaps unexpected, and surprisingly in line with computed data for **3U^−^** and **Th3^−^**.^12^ Indeed, the data for these three complexes are remarkably similar overall, with the exceptions of the Mayer bond orders that are surprisingly higher for U and Th compared to Zr. The bond orders follow the trend U>Th>Zr, and the % M contributions to the M=P bonds for U and Zr are around twice that of Th. We thus conclude that the Zr=P bond reported herein is essentially as covalent as its U=P and Th=P counterparts, and may be even less covalent; this challenges traditional views of the levels of covalency in the chemical bonding of the transition‐metals, even early d‐block ions, versus the f‐block.

To conclude, we have prepared a terminal parent zirconium‐phosphanide complex, which is a rare example of a parent d‐block phosphanide. We have used this phosphanide complex to prepare the first example of a structurally authenticated transition‐metal terminal parent phosphinidene complex under ambient conditions on bulk scale, adding to the generally rare family of early transition‐metal phosphinidene compounds, and very rare occurrences of an isolable parent phosphinidene outside of spectroscopic experiments. The zirconium phosphinidene complex reported herein appears to exhibit a weak agostic‐type Zr⋅⋅⋅HP interaction that has also been suggested to be present in cryogenic matrix isolation data on H_2_Zr=PH. Since the parent phosphinidene is free from sterically demanding substituents that may dictate the geometry of this unit, this suggests that this agostic interaction may be an intrinsic feature for Group 4 metals. Quantum chemical calculations suggest that the Zr=P bond is qualitatively about as covalent as isostructural U=P and Th=P bonds, and may even be less covalent. This is surprising, because it runs against expectations of the general view that d‐block metals, on a like‐for‐like basis, are usually expected to engage in more covalent bonding in metal–ligand complexes than for f‐block elements.

## Conflict of interest

The authors declare no conflict of interest.

## Supporting information

As a service to our authors and readers, this journal provides supporting information supplied by the authors. Such materials are peer reviewed and may be re‐organized for online delivery, but are not copy‐edited or typeset. Technical support issues arising from supporting information (other than missing files) should be addressed to the authors.

Supplementary
