# Supplementary material for: Terminal Parent Phosphanide and Phosphinidene Complexes of Zirconium(IV)
Source: Angew Chem Int Ed Engl. 2017 May 26;56(26):7669–73. doi: 10.1002/anie.201703870 (PMC5575506; doi:10.1002/anie.201703870)
Supplement: Supplementary file 1 — Supplementary [file ANIE-56-7669-s001.pdf]

Angewandte Chemie
Eine Zeitschrift der Gesellschaft Deutscher Chemiker

Supporting Information

Terminal Parent Phosphanide and Phosphinidene Complexes of Zirconium(IV)

Hannah Stafford, Thomas M. Rookes, Elizabeth P. Wildman, Gábor Balázs, Ashley J. Wooles, Manfred Scheer, and Stephen T. Liddle**

anie_201703870_sm_miscellaneous_information.pdf

Experimental

General

All manipulations were carried out using Schlenk techniques, or an MBraun UniLab glovebox, under an atmosphere of dry nitrogen. Solvents were dried by passage through activated alumina towers and degassed before use or were distilled from calcium hydride. All solvents were stored over potassium mirrors except ethers which were stored over activated 4 Å sieves. Deuterated solvent was distilled from potassium, degassed by three freeze-pump-thaw cycles and stored under nitrogen. [Zr(Tren^{DMBS})(Cl)] [**Zr1**; Tren^{DMBS} = {N($CH_2CH_2NSiMe_2Bu^t$)$_3$}], $NaPH_2$, and [$KCH_2C_6H_5$] were prepared as described previously.[1-3] Benzo-15-crown-5 ether (B15C5) was dissolved in ether, dried over 4 Å sieves, decanted and solvent removed prior to use.

^{1}H, ^{13}C, ^{29}Si, and ^{31}P NMR spectra were recorded on a Bruker 400 spectrometer operating at 400.2, 100.6, 79.5, and 162.0 MHz respectively; chemical shifts are quoted in ppm and are relative to TMS (^{1}H, ^{13}C, ^{29}Si) and 85% H_3PO_4 (^{31}P). FTIR spectra were recorded on a Bruker Tensor 27 spectrometer. UV/Vis/NIR spectra were recorded on a Perkin Elmer Lambda 750 spectrometer. Data were collected in 1mm path length cuvettes loaded in an MBraun UniLab glovebox and were run versus the appropriate reference solvent. CHN microanalyses were carried out by Mr M Jennings at the micro analytical service at the University of Manchester.

Preparation of [Zr(Tren^{DMBS})PH_2] (Zr2)

THF (20 ml) was added to a pre-cooled (−78 °C) mixture of **Zr1** (2.45 g, 4.0 mmol) and $NaPH_2$ (0.24 g, 4.4 mmol). The resulting pale orange slurry was allowed to warm to room temperature and stirred for 40 hours to afford a pale orange solution. The solvent was

removed *in vacuo* and the product was extracted into pentane and filtered yielding a brown solution. Removal of pentane afforded a dark yellow solid. Yield: 1.33 g, 54.5%. Crystals suitable for X-ray diffraction experiments were obtained from a concentrated pentane solution (2 ml) stored at −30 °C for 16 hrs. ^{1}H NMR (C_6D_6, 298 K): δ 0.32 (s, 18H, SiC*H*$_3$), 1.08 (s, 27H, SiC(C*H*$_3$)$_3$), 1.63 (d, J_{PH} = 166.3 Hz, 2H, P*H*$_2$), 2.12 (t, $^{3}J_{HH}$ = 4.77 Hz, 6H, C*H*$_2$CH$_2$), 3.31 (t, $^{3}J_{HH}$ = 4.95 Hz, 6H, CH$_2$C*H*$_2$). ^{13}C{^{1}H} NMR (C_6D_6, 298 K): δ −3.27 (Si(*C*H$_3$)$_3$), 21.32 (*C*(CH$_3$)$_3$), 27.35 (C(*C*H$_3$)$_3$), 48.38 (CH$_2$*C*H$_2$), 63.42 (*C*H$_2$CH$_2$). ^{29}Si{^{1}H} NMR (C_6D_6, 298 K): δ 2.46 (s, *Si*(CH$_3$)$_2$C(CH$_3$)$_3$). ^{31}P NMR (C_6D_6, 298 K): δ −175.28 (t, J_{PH} = 166.6 Hz, *P*H$_2$). FTIR ($\tilde{v}$, cm^{-1}): 2951 (w), 2924 (w), 2887 (w), 2849 (w), 2288 (br), 1463 (w), 1256 (w), 1049 (m), 925 (s), 799 (s), 778 (s), 723 (s), 663 (m), 567 (m), 471 (m). Anal. calc'd for $C_{24}H_{59}N_4PSi_3Zr{\cdot}0.2C_5H_{12}$: C, 48.07; H, 9.91; N, 8.97. Found: C, 47.68; H, 10.33; N, 9.14.

Preparation of [Zr(Tren^{DMBS})(PH)][K(B15C5)$_2$] (Zr3)

A solution of benzyl potassium (0.07 g, 0.52 mmol) and benzo-15-crown-5 (0.28 g, 1.04 mmol) in THF (10 ml) was added to a cold (−78 °C) solution of **Zr2** (0.32 g, 0.52 mmol) in THF (10 ml). The resulting pale orange slurry was allowed to warm to room temperature and stirred for 16 hours to afford a dark orange solution. Volatiles were removed *in vacuo* and the resulting dark orange solid was recrystallised from a mixture of toluene and HMDSO (3 ml and 0.5 ml). Yield: 0.15 g, 24.0%. Single crystals suitable for X-ray diffraction experiments were grown from a concentrated toluene/HMDSO solution stored at 5 °C for 16 hrs. ^{1}H NMR (C_6D_6, 298 K): δ 0.78 (s, 18H, SiC*H*$_3$), 1.49 (s, 27H, SiC(C*H*$_3$)$_3$), 2.38 (t, $^{3}J_{HH}$ = 4.77 Hz, 6H, CH$_2$C*H*$_2$), 3.3 – 3.56 (m, 32H, CH$_2$C*H*$_2$O), 3.58 (t, $^{3}J_{HH}$ = 4.27 Hz, 6H, C*H*$_2$CH$_2$), 6.51 (m, 4H, Ar*H*), 6.80 (m, 4H, Ar*H*), 8.53 (d, $^{1}J_{HP}$ = 173.42 Hz, 1H, P*H*). ^{13}C{^{1}H} NMR (C_6D_6, 298 K): δ −0.37 (Si(*C*H$_3$)$_3$), 22.53 (*C*(CH$_3$)$_3$), 29.18 (C(*C*H$_3$)$_3$), 48.17 (*C*H$_2$CH$_2$), 62.23

(CH_2CH_2), 68.20 (OCH_2), 68.85 (OCH_2), 69.44 (OCH_2), 70.12 (OCH_2), 114.24 (Ar-*C*H), 121.97 (Ar-*C*H), 148.99 (Ar-*C*). $^{29}Si\{^1H\}$ NMR (C_6D_6, 298 K): δ 0.32 (s, *Si*($CH_2)_2C(CH_3)_3$). ^{31}P NMR (C_6D_6, 298 K): δ 246.75 (br, FWHM; 255.87 Hz, *P*H). FTIR ($\tilde{\nu}$, cm^{-1}): 2948 (w), 2925 (w), 2876 (w), 2837 (w), 2100 (vw), 1596 (w), 1505 (m), 1453 (w) 1409 (w), 1256 (s), 1122 (s), 1094 (s), 1044 (s), 934 (s), 819 (s), 804 (s), 742 (s), 673 (m), 559 (m), 459 (m). Anal. calc'd for $C_{52}H_{98}KN_4O_{10}PSi_3Zr{\cdot}0.8\ C_6H_{18}OSi_2$: C, 51.89; H, 8.62; N, 4.26. Found: C, 52.05; H, 8.76; N, 4.10.

UV/Vis/NIR Electronic Absorption spectra of Zr(2) (top) and Zr(3) (bottom) in toluene

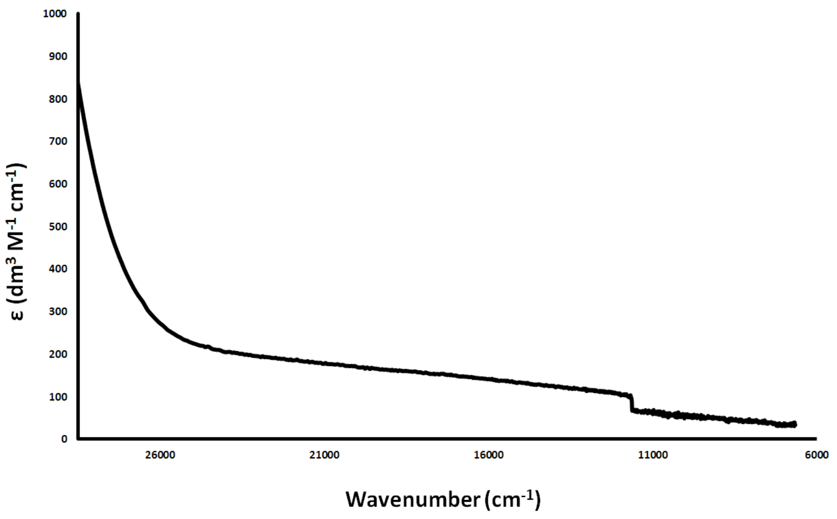

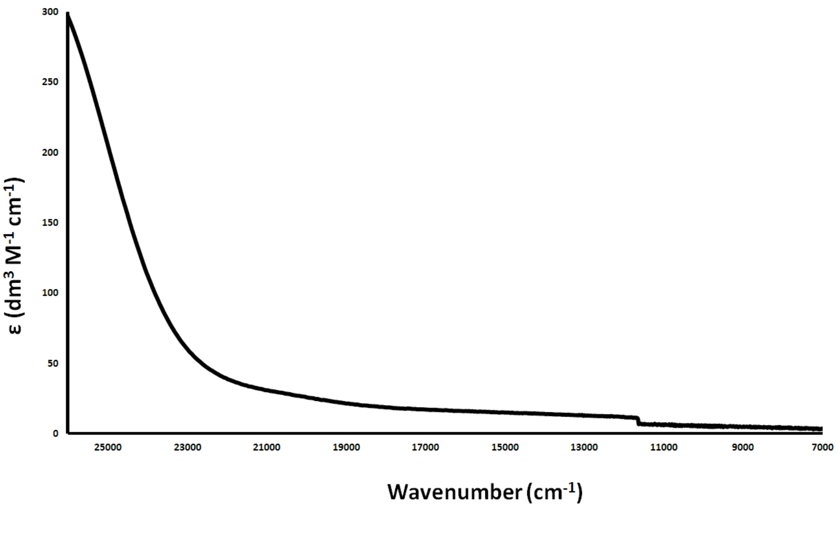

Table S1. Experimental X-ray crystallographic details for Zr2 and Zr3.

	**Zr2**	**Zr3**
Formula	$C_{24}H_{59}N_4PSi_3Zr$	$C_{52}H_{98}KN_4O_{10}PSi_3Zr$
Fw	610.21	1184.90
Cryst size, mm^3	0.243 x 0.190 x 0.131	0.550 x 0.191 x 0.150
Cryst syst	Orthorhombic	Monoclinic
Space group	*P*bca	$P2_1$/n
a, Å	19.9784(10)	22.115(2)
b, Å	15.4052(9)	13.0823(10)
c, Å	22.7205(13)	24.036(2)
α, °	90	90
β, °	90	115.107(14)
γ, °	90	90
V, $Å^3$	6992.7(7)	6296.7(12)
Z	8	4
ρ_{calc} g cm^{-3}	1.159	1.250
μ, mm^{-1}	0.481	0.376
no. of reflections measd	30087	36879
no. of unique reflns, Rint	6160, 0.1582	11090, 0.10902
no. of reflns with $F^2 > 2s(F^2)$	3623	8229
transmn coeff range	0.921-0.959	0.880-0.958
R, $R_w{}^a$ ($F^2 > 2s(F^2)$)	0.0754, 0.1513	0.0942, 0.2372
R, $R_w{}^a$ (all data)	0.1291, 0.1729	0.1174, 0.2616
S^a	1.033	1.084
Parameters	399	667
max.,min. diff map, e $Å^{-3}$	0.857, -0.856	2.907, -1.469

Table S2. Selected bond lengths (Å) and angles (°) for Zr2 and Zr3.

	**Zr2**		
Zr1-P1	2.690(2)	Zr1-N1	2.060(4)
Zr1-N2	2.056(5)	Zr1-N3	2.069(4)
Zr1-N4	2.516(5)	P1-H1A	1.425(10)
P1-H1B	1.424(10)	H1A-P1-H1B	93.6(8)
N1-Zr1-P1	105.68(14)	N1-Zr1-N3	112.72(19)
N1-Zr1-N4	74.49(17)	N2-Zr1-P1	105.45(14)
N2-Zr1-N1	112.74(18)	N2-Zr1-N3	112.50(19)
N2-Zr1-N4	73.37(17)	N3-Zr1-P1	107.04(14)
N3-Zr1-N4	73.96(17)	N4-Zr1-P1	178.73(13)
Zr1-P1-H1A	101.3(6)	Zr1-P1-H1B	101.2(6)
	**Zr3**		
Zr1-P1	2.4723(17)	Zr1-N1	2.135(5)
Zr1-N2	2.109(5)	Zr1-N3	2.127(5)
Zr1-N4	2.586(4)	Zr1-H1	2.322(19)
P1-H1	1.460(10)		
K1-O1	2.970(4)	K1-O2	2.904(4)
K1-O3	2.757(4)	K1-O4	2.840(4)
K1-O5	2.909(4)	K1-O6	2.883(4)
K1-O7	2.853(4)	K1-O8	2.785(4)
K1-O9	2.826(4)	K1-O10	2.965(4)
P1-Zr1-N4	179.76(12)	P1-Zr1-H1	35.3(3) .
N1-Zr1-P1	107.92(13)	N1-Zr1-N4	72.31(15)
N1-Zr1-H1	77.5(5)	N2-Zr1-P1	107.30(13)
N2-Zr1-N1	107.64(19)	N2-Zr1-N3	110.19(17)
N2-Zr1-N4	72.58(16)	N2-Zr1-H1	100.7(7)
N3-Zr1-P1	107.87(13)	N3-Zr1-N1	115.61(18)
N3-Zr1-N4	72.01(16)	N3-Zr1-H1	139.2(5)

Density Functional Theory Calculations

General

Restricted geometry optimisations were performed for the full models of **Zr2** and the anion component of **Zr3** (**Zr3**$^-$) using coordinates derived from the X-ray crystal structures. No constraints were imposed on the structures during the geometry optimisations. The calculations were performed using the Amsterdam Density Functional (ADF) suite version 2012.01.[4,5] The DFT geometry optimisations employed Slater type orbital (STO) triple-ζ-plus polarisation all-electron basis sets (from the ZORA/TZP database of the ADF suite). Scalar relativistic approaches were used within the ZORA Hamiltonian for the inclusion of relativistic effects and the local density approximation (LDA) with the correlation potential due to Vosko et al[6] was used in all of the calculations. Gradient corrections were performed using the functionals of Becke[7] and Perdew.[8] MOLEKEL[9] was used to prepare the three-dimensional plot of the electron density. Natural Bond Order (NBO) analyses were carried out with NBO 5.0.[10] The Atoms in Molecules analysis[11,12] was carried out with Xaim-1.0.[13]

Selected Kohn Sham and NBO Orbitals for Zr2

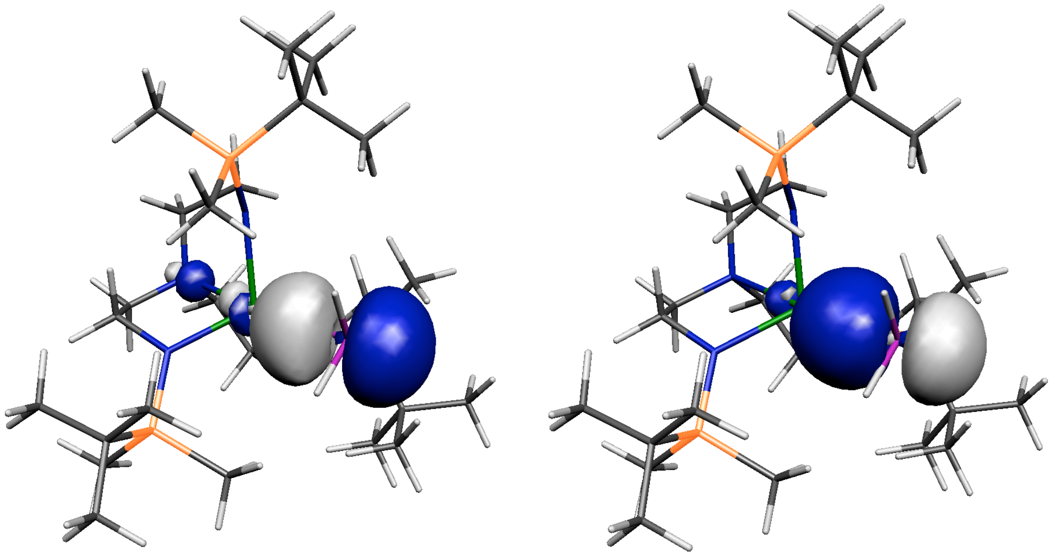

Left: HOMO (164, –4.655 eV). Right: NBO representation of the Zr-P σ-bond.

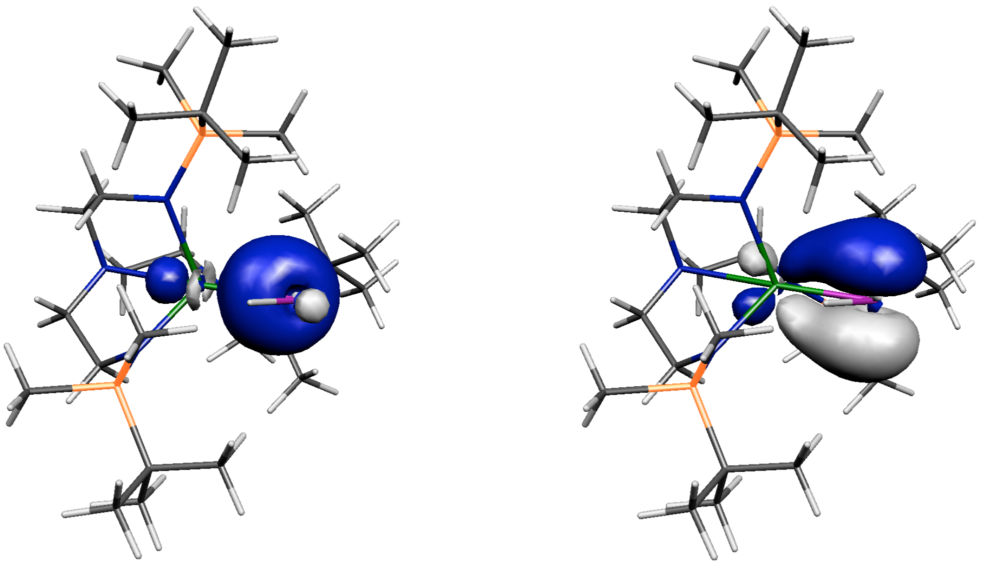

Left: NBO representation of the Zr-P σ-bond. Right: NBO representation of the Zr-P π-bond.

Final Coordinates and Single Point Energy of Zr2 After Geometry Optimisation

1.C	2.763090	0.014749	-3.893502
2.C	-0.234003	-1.805730	-2.665710
3.C	-2.114946	-0.219023	-2.400499
4.C	5.731710	0.131105	-2.044271
5.C	-1.465989	1.161970	-2.266017
6.C	-1.696647	4.740704	-2.072285
7.C	4.661315	-2.136119	-1.863315
8.C	1.081800	-2.005012	-1.905781
9.C	4.558544	-0.659545	-1.419285
10.C	2.803847	1.942136	-1.534626
11.C	-2.037200	-2.278204	-1.035347
12.C	-3.994886	2.506063	-0.631483
13.C	-1.580490	4.496566	-0.550745
14.C	-0.108640	4.684820	-0.127794
15.C	4.673061	-0.594174	0.117785
16.C	-2.450650	5.539867	0.187683
17.C	-2.304758	-1.807791	0.396924
18.C	-0.936869	-4.374315	1.864601
19.C	-2.141023	2.490905	1.789037
20.C	1.372632	-2.537225	2.559969
21.C	-2.879654	-2.506397	3.967323
22.C	-1.373605	-2.166294	4.034730
23.C	-1.215295	-0.667877	4.365821
24.C	-0.721238	-2.996918	5.163687
25.H	3.638009	0.501247	-4.353292
26.H	2.719995	-1.012188	-4.286557
27.H	1.868801	0.549566	-4.250710
28.H	-2.316889	-0.477629	-3.457669

29.H	-0.055750	-1.081161	-3.471376
30.H	5.710168	0.113050	-3.145545
31.H	4.581539	-2.250132	-2.955950
32.H	-0.586732	-2.746540	-3.129664
33.H	-0.536752	1.191778	-2.861873
34.H	-2.138874	1.904669	-2.724847
35.H	-1.048560	4.062948	-2.647547
36.H	6.694502	-0.310913	-1.729628
37.H	-2.728635	4.617738	-2.436345
38.H	1.837092	-2.387698	-2.611570
39.H	-1.386870	5.772801	-2.316756
40.H	5.638313	-2.555989	-1.563016
41.H	-3.077053	-0.195508	-1.871680
42.H	3.584271	2.491525	-2.083893
43.H	5.736406	1.184393	-1.722744
44.H	-4.144409	2.565935	-1.719877
45.H	-2.973026	-2.520248	-1.574646
46.H	3.883522	-2.758136	-1.395911
47.H	1.834496	2.381526	-1.817398
48.H	0.952756	-2.795461	-1.145847
49.H	-1.430549	-3.192372	-0.985557
50.H	-4.621330	3.288464	-0.174697
51.H	0.553944	3.962954	-0.626361
52.H	0.238884	5.699182	-0.394223
53.H	-4.387521	1.535020	-0.290955
54.H	2.952110	2.131141	-0.462760
55.H	-2.109206	6.560657	-0.061424
56.H	-3.512656	5.474647	-0.095569
57.H	5.619193	-1.057138	0.450725
58.H	4.668655	0.441337	0.488979
59.H	3.848961	-1.129173	0.611911
60.H	-2.971404	-0.927664	0.376377
61.H	0.028850	4.566903	0.957487
62.H	-0.350304	-4.681679	0.984262
63.H	-2.865147	-2.595193	0.926383
64.H	-2.385267	5.432504	1.281224
65.H	-1.999869	-4.541462	1.636530
66.H	1.880976	-2.603450	1.584879
67.H	-2.345248	1.446066	2.070308
68.H	2.599892	1.589905	1.505322
69.H	-2.924309	3.110265	2.252749
70.H	-1.181646	2.774506	2.241738
71.H	-0.670919	-5.058788	2.685808
72.H	0.844907	2.277512	2.358326
73.H	1.688890	-3.409894	3.152980
74.H	1.733526	-1.630861	3.064899
75.H	-3.398680	-1.923547	3.190704
76.H	-1.706573	-0.034537	3.612576
77.H	-3.055228	-3.574710	3.765946
78.H	-0.159731	-0.359618	4.418737

79.H	-3.367436	-2.269257	4.930014
80.H	-0.784582	-4.081491	4.978247
81.H	-1.679367	-0.442063	5.343001
82.H	0.339707	-2.737352	5.299740
83.H	-1.233671	-2.798813	6.122487
84.N	-1.259486	-1.243236	-1.755919
85.N	1.483575	-0.727742	-1.276435
86.N	-1.192314	1.446542	-0.839422
87.N	-1.018081	-1.485491	1.050669
88.P	1.501182	1.013027	2.214513
89.Si	2.874667	0.105269	-1.990915
90.Si	-2.177376	2.711917	-0.090433
91.Si	-0.508781	-2.579623	2.353017
92.Zr	0.076266	0.036502	0.088828

Energy: –492.01578420 eV

Final Coordinates and Single Point Energy of $Zr3^{-}$ After Geometry Optimisation

1.C	2.347394	-2.654162	-4.825652
2.C	1.647329	-0.243522	-4.750532
3.C	-0.895694	-2.641318	-3.939772
4.C	1.985241	-1.479722	-3.890755
5.C	3.206446	-1.149639	-3.007394
6.C	-1.107017	0.300294	-2.614351
7.C	-2.490677	0.342881	-1.954953
8.C	0.951375	-3.367815	-1.626978
9.C	2.469830	2.702908	-1.377683
10.C	0.299283	4.825839	-1.145875
11.C	-2.369732	2.269792	-0.411144
12.C	-3.307575	0.121286	0.363105
13.C	2.834531	5.507547	0.878729
14.C	-2.706674	-1.209983	0.817414
15.C	-1.332260	2.685740	0.638199
16.C	2.106568	4.217338	1.315120
17.C	3.144640	3.217422	1.868957
18.C	-1.889158	-4.294384	2.113230
19.C	1.099851	4.562144	2.432288
20.C	0.528827	-3.870418	2.627081
21.C	-0.917581	-3.589610	3.084001
22.C	-2.906584	-1.401869	4.031726
23.C	0.071536	-0.799168	4.108005
24.C	-1.118332	-4.176594	4.498099
25.H	1.530157	-2.895771	-5.524251
26.H	3.238164	-2.401014	-5.432626
27.H	2.507235	0.028212	-5.392389
28.H	0.785578	-0.420420	-5.414874
29.H	-1.144736	-1.982361	-4.786421
30.H	2.588076	-3.568278	-4.260151

31.H	-0.574147	-3.606890	-4.361681
32.H	1.415898	0.628414	-4.121253
33.H	4.065782	-0.847109	-3.637393
34.H	-1.252241	-0.008382	-3.664868
35.H	-1.823740	-2.823233	-3.373644
36.H	-0.712501	1.333690	-2.662698
37.H	3.521771	-2.012323	-2.401783
38.H	-3.192301	0.984681	-2.531148
39.H	2.993965	-0.331353	-2.305933
40.H	1.992091	2.315260	-2.290543
41.H	1.396322	-4.200367	-2.196357
42.H	-2.896197	-0.678775	-1.950817
43.H	-0.289873	4.415158	-1.981665
44.H	3.186416	3.484231	-1.678815
45.H	1.043574	5.515091	-1.575577
46.H	0.067899	-3.755873	-1.096225
47.H	-2.074574	2.710734	-1.373362
48.H	3.038256	1.877616	-0.926744
49.H	1.667129	-3.021004	-0.865136
50.H	-0.381130	5.428328	-0.523382
51.H	3.541249	5.322368	0.053369
52.H	-4.306811	-0.021654	-0.102440
53.H	-3.382799	2.651931	-0.161101
54.H	-2.618278	-1.876422	-0.062582
55.H	2.132470	6.290375	0.549533
56.H	2.369025	0.203261	0.054928
57.H	3.944554	3.019551	1.138875
58.H	-1.377599	3.785347	0.741819
59.H	-1.747624	-3.942294	1.081552
60.H	3.420337	5.921738	1.721680
61.H	-3.436293	0.756189	1.251218
62.H	-3.442899	-1.697676	1.480414
63.H	-1.646308	2.289576	1.622928
64.H	0.742634	-3.432946	1.640869
65.H	-1.715165	-5.387492	2.119511
66.H	0.333423	5.280017	2.094619
67.H	-2.944807	-4.129719	2.386266
68.H	2.693074	2.246114	2.124558
69.H	3.623445	3.626287	2.779751
70.H	0.706187	-4.961567	2.563394
71.H	0.585298	3.660340	2.793977
72.H	1.621842	5.020958	3.293779
73.H	1.267128	-3.454962	3.329384
74.H	-3.740044	-2.032301	3.683439
75.H	1.039775	-0.773490	3.583771
76.H	-3.224720	-0.349693	3.948942
77.H	-0.222370	0.244120	4.306656
78.H	-0.881277	-5.258053	4.499444
79.H	-2.156288	-4.066597	4.850515
80.H	-2.761168	-1.618232	5.102665

81.H	-0.458207	-3.698401	5.239591
82.H	0.199136	-1.302792	5.080921
83.N	-0.188894	-0.586852	-1.878641
84.N	-2.382364	0.801277	-0.557690
85.N	0.006898	2.192996	0.266010
86.N	-1.401101	-0.995237	1.469228
87.P	2.214689	-0.874367	1.032653
88.Si	0.461770	-1.944635	-2.769657
89.Si	1.173271	3.408117	-0.187120
90.Si	-1.256230	-1.670398	3.080039
91.Zr	0.058909	0.006720	0.202174

Energy: −489.69390901 eV

References

1. C. Morton, I. J. Munslow, C. J. Sanders, N. W. Alcock, P. Scott, *Organometallics* **1999**, *18*, 4608.
2. R. Klement, G. Brauer, *Handbuch der Präparativen Anorganischen Chemie*, 3rd Ed, Ferdinand Enke Verlag, Stuttgart, **1975**, Issue 1, pp 516
3. P. J. Bailey, R. A. Coxall, C. M. Dick, S. Fabre, L. C. Henderson, C. Herber, S. T. Liddle, D. Loroño-González, A. Parkin, S. Parsons, *Chem. Eur. J.* **2003**, *9*, 4820.
4. C. Fonseca Guerra, J. G. Snijders, G. te Velde E. J. Baerends, *Theor. Chem. Acc.* **1998**, *99*, 391.
5. G. te Velde, F. M. Bickelhaupt, S. J. A. van Gisbergen, C. Fonseca Guerra, E. J. Baerends, J. G. Snijders T. Ziegler, *J. Comput. Chem.* **2001**, *22*, 931.
6. S. H. Vosko, L. Wilk, M. Nusair, *Can. J. Phys.* **1980**, *58*, 1200.
7. A. D. Becke, *Phys. Rev. A.* **1988**, *38*, 3098.
8. J. P. Perdew, *Phys. Rev. B.* **1986**, *33*, 8822.
9. S. Portmann, H. P. Luthi, *Chimia* **2000**, *54*, 766.
10. NBO 5.0: E. D. Glendening, J. K. Badenhoop, A. E. Reed, J. E. Carpenter, J. A. Bohmann, C. M. Morales, F. Weinhold, (Theoretical Chemistry Institute, University of Wisconsin, Madison, WI, 2001); http://www.chem.wisc.edu/~nbo5.
11. R. F. W. Bader, *Atoms in Molecules: A Quantum Theory*, Oxford University Press, New York, 1990.
12. R. F. W. Bader, *J. Phys. Chem. A.* **1998**, *102*, 7314.
13. http://www.quimica.urv.es/XAIM.
